# Quinoline-Based Hybrid Compounds with Antimalarial Activity

**DOI:** 10.3390/molecules22122268

**Published:** 2017-12-19

**Authors:** Xhamla Nqoro, Naki Tobeka, Blessing A. Aderibigbe

**Affiliations:** Department of Chemistry, University of Fort Hare, Alice Campus, Alice 5700, Eastern Cape, South Africa; xnqoro@gmail.com (X.N.); tobekanaki@gmail.com (N.T.)

**Keywords:** 4-aminoquinoline, 8-aminoquinoline, malaria, infectious disease, hybrid compound

## Abstract

The application of quinoline-based compounds for the treatment of malaria infections is hampered by drug resistance. Drug resistance has led to the combination of quinolines with other classes of antimalarials resulting in enhanced therapeutic outcomes. However, the combination of antimalarials is limited by drug-drug interactions. In order to overcome the aforementioned factors, several researchers have reported hybrid compounds prepared by reacting quinoline-based compounds with other compounds via selected functionalities. This review will focus on the currently reported quinoline-based hybrid compounds and their preclinical studies.

## 1. Introduction

Malaria is a parasitic infectious disease that is a threat to approximately half of the world’s population. This parasitic disease is transmitted to humans by a female *Anopheles* mosquito when it takes a blood meal. Pregnant women and young children in the sub-tropical regions are more prone to malaria infection. In 2015, 214 million infections were reported, with approximately 438,000 deaths and the African region accounted for 90% of the deaths [1,2,3]. Malaria is caused by five parasites of the genus *Plasmodium*, but the majority of deaths are caused by *Plasmodium falciparum* and *Plasmodium vivax* [1,4,5,6]. Effective drugs to treat malaria have been developed and they include drugs such as quinine, quinoline, mefloquine, and artemisinin. Quinoline was the drug of choice for the treatment of malaria caused by *Plasmodium falciparum.* However, the parasite has developed resistance to the drug. To overcome drug resistance, different approaches which include combination therapy have been evaluated. More than a decade ago, the combination therapy which involves the combination of two or more antimalarials has been reported to be the future approach to overcome drug resistance of antimalarials. Hybridization approach has also been utilized, it involves the combination of two distinctive pharmacophoric features from new and known drugs to form a molecule with enhanced efficacy against most of the malaria stages [5,7]. Antimalarial drugs containing 4-aminoquinoline scaffolds are good precursors for hybridization with other antimalarials and metal-based compounds via selected functionalities [8]. Hybrid compounds are more effective compared to the multi-component drugs because of the lower occurrence of drug-drug adverse effects [9]. This review will be focused on the synthesis, in vitro and in vivo studies of hybrid compounds containing 4- and 8-aminoquinoline derivatives.

## 2. Classes of Antimalarial Drugs

**Figure 1:**
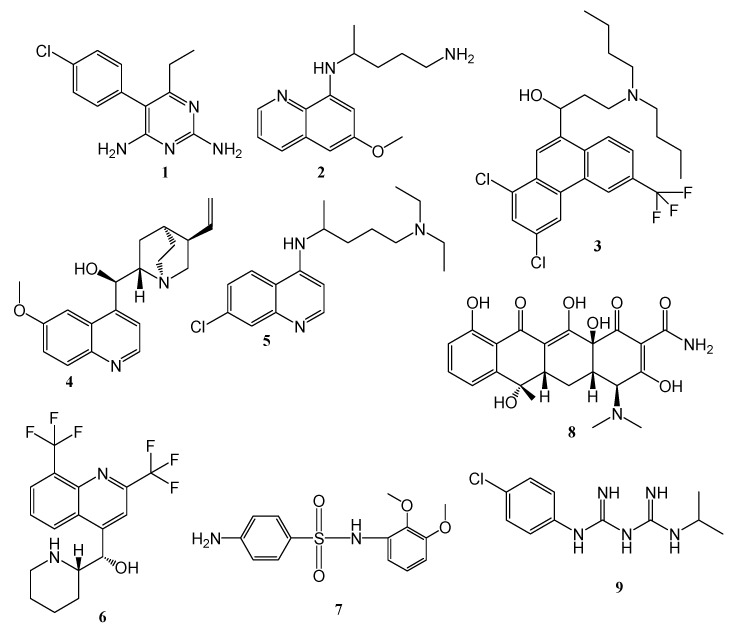
Examples of antimalarials classified based on their activity against malaria life stage.

Antimalarials are classified based on their activities against the malaria life stages within the human host. This classification includes [10,11] (Figure 1): The tissue schizontocidal drugs which can be classified as causal drugs and they hinder erythrocytic stages such as pyrimethamine **1** and primaquine **2**, which also prevent a relapse caused by hypnozoites of *P. vivax* and *P. ovale* in the liver; hypnozoitocidal drugs act against exo-erythrocytic hypnozoites of *P. vivax* and *P. ovale* such as primaquine; blood schizontocidal drugs act on the blood forms of the parasite thereby eliminating clinical attacks of malaria. These include halofantrine (**3**), quinine (**4**), chloroquine (**5**), mefloquine (**6**), pyrimethamine, sulfones, sulfadoxine (**7**) tetracycline (**8**). Gametocytocides such as primaquine, chloroquine and quinine prevent gametocyte forms in the blood, thereby hindering mosquito infection and sporontocides such as primaquine, chloroguanide (**9**) and pyrimethamine hinder the development of oocyst and multiplication of the parasites in the mosquito gut when it takes a blood meal from the human host.

**Figure 2:**
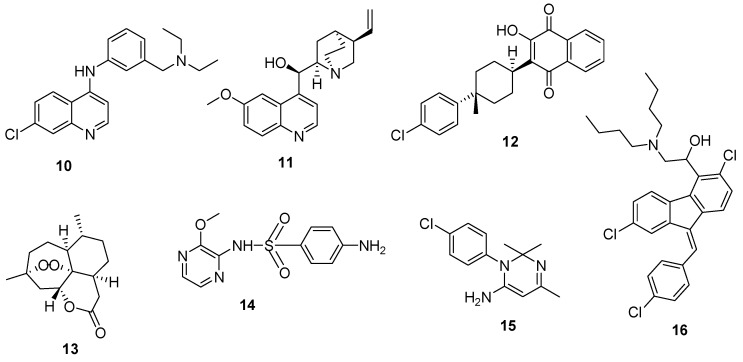
Examples of antimalarials classified based on their structure.

Antimalarial drugs are also classified based on their structures into groups such as 4-aminoquinolines, like chloroquine and amodiaquine (**10**); 8-aminoquinolines such as primaquine; 4-quinolinemethanols, e.g., mefloquine; quinoline-containing cinchona alkaloids, e.g., quinine and quinidine (**11**); hydroxynaphthoquinones, e.g., atovaquone (**12**); diaminopyrimidines such as pyrimethamine; sesquiterpene-lactones such as artemisinin (**13**), arteether, artemether; sulfonamides, e.g., sulfalene (**14**), sulfadoxine, dapsone; biguanides such as proguanil, chlorproguanil and cycloguanil (**15**); antibiotics such as tetracycline, azithromycin and doxycycline and phenanthrene-methanols such as lumefantrine (**16**) [11,12] (Figure 2). Each of these classes has its own unique mode of action and mechanism of resistance against the malaria parasite. 

## 3. Mechanism of Antimalarial Drug Resistance

### 3.1. Mechanism of Resistance in Quinolines

The mechanism of resistance in quinolines such as chloroquine is not well understood. Many researchers have tried to explain the possible mechanisms of resistance in quinolones. Chloroquine in an uncharged form diffuses freely into the erythrocyte onto the digestive vacuole where it becomes protonated. In the digestive vacuole, it binds to the haematin, a toxic byproduct of the haemoglobin proteolysis [13]. Chloroquine accumulates significantly in chloroquine sensitive parasites compared to the chloroquine resistant strains [13]. The resistance of chloroquine is associated with an increased level of drug efflux. Chloroquine resistant strains have been reported to release the drug at a faster rate compared to the chloroquine sensitive parasites, resulting in drug resistance [14]. Reduced affinity of chloroquine for heme resulting in reduced chloroquine uptake also contribute to chloroquine resistance [15]. *P. falciparum* chloroquine resistance transporter (PfCRT) also contributes to the resistance of parasite to chloroquine drug [16]. 

### 3.2. Mechanism of Resistance in Artemisinins

The resistance of plasmodium parasites to artemisinin is attributed to young ring forms which enter a quiescence state after exposure to artemisinin and then resume growth after the removal of the artemisinin [13,17]. This process is influenced by the gene, Pfk13 [13,17]. Its poor bioavailability also enhances the development of drug resistance of the plasmodium parasites.

### 3.3. Mechanism of Resistance to 4-Quinolinemethanols 

Resistance to 4-quinolinemethanols such as mefloquine is attributed to an overexpression of Pgh-1 that enhances the efflux of mefloquine out of the food vacuole of the parasite [18]. However, resistance to mefloquine is also possible in the absence of pfmdr1 amplification [19]. 

### 3.4. Mechanism of Resistance to Hydroxynaphthoquinones and Diaminopyrimidines

Resistance of plasmodium parasites to hydroxynaphthoquinones such as atovaquone is reported to be rapid and is due to a single-point mutation in the cytochrome-b gene [20]. Resistance of plasmodium parasites to diaminopyrimidine-based antimalarials such as pyrimethamine is due to the mutations in the gene for dihydrofolate reductase. These mutations reduce the binding affinity between pyrimethamine and dihydrofolate reductase by the loss of interactions which are steric and from hydrogen bonds [21].

### 3.5. Mechanism of Resistance to Sulphonamides 

Studies have reported that mutations at five DHPS codons (S436A/F; A437G; K540E; A581G; and A613S/T) contribute to sulfadoxine resistance in *P. falciparum* [22]. The mutant DHPS enzyme decreased affinity for sulfadoxine and contributes to its resistance in plasmodium parasites [22]. 

Drug resistance that is common in most antimalarials is as a result of spontaneous mutations that decrease the sensitivity to the drugs. In some antimalarials, a single point mutation produces resistance whereas other classes of antimalarial may require multiple mutations that result in drug resistance [23]. There are other factors that contribute to drug resistance in antimalarials such as poor patients’ compliance, drug mismatch, drug-drug interactions, presumptive treatment of malaria and low drug quality [23]. In order to overcome drug resistance in malaria treatment, antimalarials are combined, resulting in enhanced efficacy. However, the combination of two or more drugs can also result in pharmacological interactions between the drugs, resulting in toxicity and drug mismatch [24].

## 4. Hybrid Compounds 

The design of hybrid compounds with antimalarial activity offers unique advantages such as cost effectiveness and reduced risk of drug-drug interaction. Hybrid drugs are distributed, metabolized, and excreted at a single rate and there is no competition for plasma protein binding that can result in drug interactions. The linker controls the pharmacokinetics behaviour of the drugs [25]. According to Muregi and Ishih, they can be classified as conjugates in which the pharmacophores are separated by a linker group that is distinct; cleavage conjugates in which the pharmacophores are separated by a metabolized linker; fused hybrid molecules with reduced linker between pharmacophores, resulting in the closeness of the pharmacophores and merged hybrid in which the framework is merged [26]. 

### 4.1. Quinoline–Artemisinin Derivatives Hybrid Compounds

**Figure 3:**
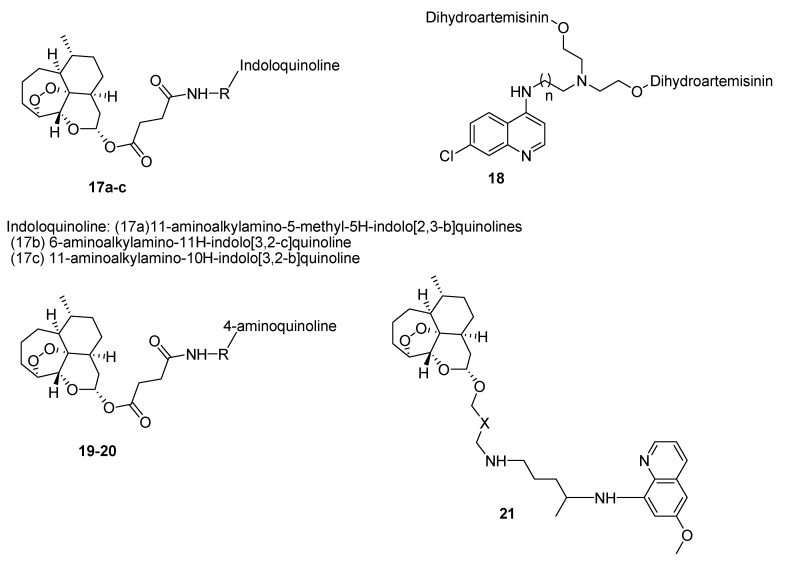
Quinoline-artemisinin hybrids.

There are reports of quinoline-artemisinin hybrids with good antimalarial activity (Figure 3). Wang et al. synthesized artesunate-quinoline hybrid compounds **17** with enhanced antimalarial activity when compared to the individual drugs. They were good β-haematin inhibitors in vivo in mice at a dosage of 10 mg kg^−1^ once a day over a period of four days, resulting in a significant reduction of parasitemia and a mean mouse survival time of 7.7 days [27]. Lombard et al. prepared artemisinin-quinoline hybrids **18** containing different aminoquinoline moieties. However, the compounds were less active than dihydroartemisinin. The compounds showed good selectivity towards *P. falciparum* and the compound with a chain length of three carbon atoms exhibited excellent antiplasmodial activity [28]. Walsh et al. incorporated quinine carboxylic acid derivative to dihydroartemisinin via an ester linkage [29]. The compound exhibited superior activity when compared to individual drugs, indicating that the quinine and artemisinin moieties were preserved and also revealed the benefit of covalent binding of both drugs [29]. Lombard et al. prepared hybrid compounds as oxalate salts by coupling dihydroartemisinin with selected aminoquinoline moieties followed by treatment with oxalic acid to form oxalate hybrids **19** [30]. In vitro antiplasmodial activity against the chloroquine sensitive D10 strain and chloroquine resistant Dd2 strain of *P. falciparum* revealed that the oxalate hybrids exhibited better antiplasmodial activity compared to the free base hybrids but with slight toxic effects [30]. Lombard et al. also evaluated artemisinin-aminoquinoline hybrids **20** in vivo against *Plasmodium vinckei* [31]. In vivo evaluation in *Plasmodium vinckei*-infected mice by intraperitoneal or oral administration of the hybrid compound in a dosage of 0.8 mg/kg, 2.5 mg/kg, 7.5 mg/kg or 15 mg/kg over a period of four days. A complete cure of the mice was significant when a dosage of 15 mg/kg was administered intraperitoneally and also when a dosage of 50 mg/kg was administered orally [31]. Capela et al. prepared hybrids containing primaquine and dihydroartemisinin pharmacophoric units **21** [32]. In vitro and in vivo efficacy of the hybrids on *Plasmodium* liver and the blood stage malaria showed that the hybrid compounds exhibited good antiplasmodial activity in vitro against liver stage malaria. The efficacy of primaquine was increased by introducing dihydroartemisinin into the hybrid molecules. The hybrid compounds reduced the parasite loads in the liver of the mice significantly [32]. 

### 4.2. Quinoline–Ferrocene Hybrid Compounds

**Figure 4:**
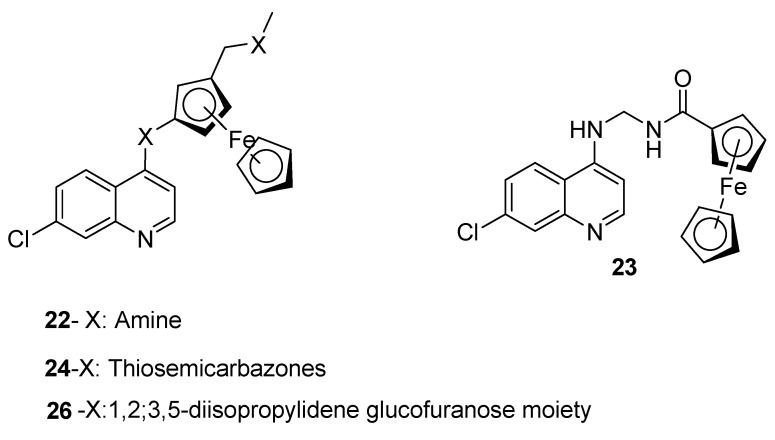

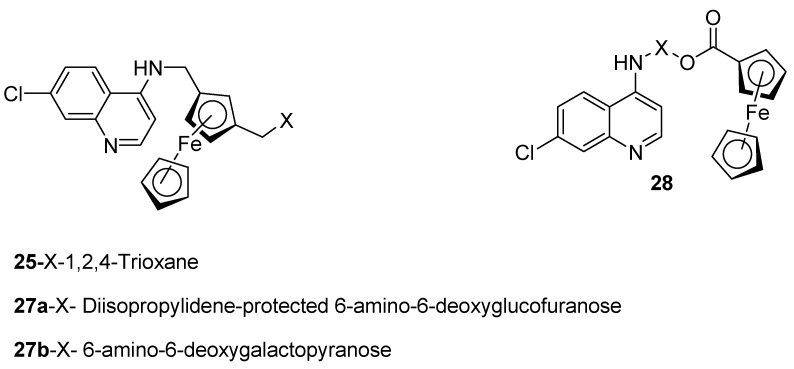
Quinoline-ferrocene hybrid compounds.

Quinoline-ferrocene hybrid compounds have also been reported to be an effective antimalarials (Figure 4). Ferroquine (**22**) has been reported to be effective against *P. falciparum* isolates which are multi-drug resistant when compared to other antimalarials such as chloroquine, piperaquine etc. [33]. However, artesunate was more effective than ferroquine [33]. Ferroquine is believed to act by blocking *Pf*CRT and acting like a resistance reversing agent due to its lipophilic properties [33,34]. Domarle et al. prepared quinoline-ferrocene analogues and the covalent binding of chloroquine to ferrocene compound inhibited resistance of the parasites. The tartaric acid analogues were very effective when compared to chloroquine drug at a low concentration [35]. Ferrocene acts as an inhibitor of chloroquine resistance without enhancing the activity of chloroquine [35]. Biot et al. synthesized ferrocene-quinoline analogue from triazacyclononane, ferrocene and aminoquinoline. The analogue exhibited potent antimalarial activity against the chloroquine-resistant strain Dd2 in vitro [36]. N’Da et al. prepared quinoline–ferrocene hybrids **23** with selected linkers from ferrocene carboxaldehyde and 4-aminoquinolines. The compounds were prepared via amination reactions of 4,7-dichloroquinolines with selected diamines [37]. Hybrids with rigid linkers were not biologically active when compared to the hybrids with flexible linkers that were effective against D10 and Dd2 strains of *Plasmodium falciparum*. The hybrid compound with a 3-aminopropyl methylamine linker was the most effective antimalarial compound with IC_50_ = 0.008 vs. 0.148 μM, i.e., 19-fold higher than the equimolar chloroquine–ferrocene combination with IC_50_ = 3.7 vs. 41 ng/mL, and tenfold more active against the Dd2 strain [37]. Biot et al. prepared derivatives of ferroquine that mimic hydroxychloroquine [38]. In vitro evaluation on Cambodian field isolates revealed a highly potent antimalarial activity of the derivatives against *P. falciparum*. The derivatives were 6-fold more active than chloroquine and 1.5 fold less active than ferroquine against all strains and isolates of *P. falciparum* [38]. The compounds were also found to be potential antimalarials for countries with co-infection of malaria with HIV and SARS [38]. Biot et al. also prepared quinoline-ferrocene hybrid compounds containing thiosemicarbazones **24** [39]. In vitro evaluation on parasite *Plasmodium falciparum* and against the parasitic cysteine protease falcipain-2 revealed that the aminoquinoline structure enhanced the transportation of the compounds to the food vacuole of the parasite and the ferrocene moiety maintained the activity of the 4-aminoquinoline [39]. Chavain et al. prepared quinoline-ferroquine compounds conjugated to a glutathione reductase inhibitor via an amide bond. The antimalarial activity of the dual molecule was unique when compared to ferroquine and chloroquine [40]. In vitro evaluation against NF54 and K1, chloroquine sensitive and resistant strains of *P. falciparum* showed a decrease in the antimalarial activity. The reduced antimalarial activity of the hybrid compounds was attributed to the cleavage of the amide bond and the side chain when metabolised in the food vacuole of the parasite, revealing that the design of the molecules influences their antimalarial activity [40]. Bellot et al. prepared trioxaferroquines composed of 1,2,4-trioxane linked covalently to ferroquine (**25**) [41]. The drug exhibited good in vitro evaluation against chloroquine-resistant strains of *P. falciparum* [41]. Herrmann et al. prepared hybrid compounds **26** composed of a ferrocene scaffold, a chloroquine derivative and a 1,2;3,5-(diisopropylidene)-α-D-glucofuranose moiety with good in vitro antiplasmodial activity against Dd2 and K1 strains of *P. falciparum* [42]. In another research report by Herrmann et al. conjugated ferrocene scaffolds via ether linker with either 7-chloroquinoline followed by incorporation of diisopropylidene-protected 6-amino-6-deoxyglucofuranose or 6-amino-6-deoxygalactopyranose by reductive amination to produce hybrid compounds **27a** and **27b** [43]. The carbohydrate moiety enhanced the antimalarial activity of the compounds with an IC_50_ = 0.77 μM [43]. The antimalarial activity was significant in chloroquine-resistant Dd2 strain when compared to chloroquine-sensitive D10 strain [43]. Sallas et al. prepared ferrocenophane derivatives of ferroquine which were active against chloroquine-sensitive and -resistant strains. The lipophilicity of these derivatives influenced their ability to overcome resistance [44]. Ferroquine is characterized by a weak base, high lipophilicity at pH 7.4, unique conformation from the present of intramolecular hydrogen bond under non-polar conditions which enhances its permeation ability across the parasite membrane, resulting in a high accumulation in the parasite digestive vacuole. It inhibits the self-assembly of the hemozoin crystal and generates reactive oxygen species, thereby inducing lipid peroxidation and the alteration of digestive vacuole [45]. The presence of the intra-molecular hydrogen bond allows ferroquine to overcome resistance mechanisms by avoiding cross-resistance [45]. David et al. also prepared 4-aminoquinoline compounds **28** conjugated to ferrocene group via ester groups. The compounds with a ferrocenylformic acid moiety were active against both chloroquine-resistant and -sensitive strains of *Plasmodium falciparum*. However, chloroquine exhibited better antimalarial activity against the chloroquine-sensitive strain. The compound with good antimalarial activity exhibited an IC_50_ of 0.13 mM on the resistant strain and was 2.5-fold higher than chloroquine with an IC_50_ = 0.34 mM [46]. 

### 4.3. Quinoline-Trioxolanes Hybrid Compounds

**Figure 5:**
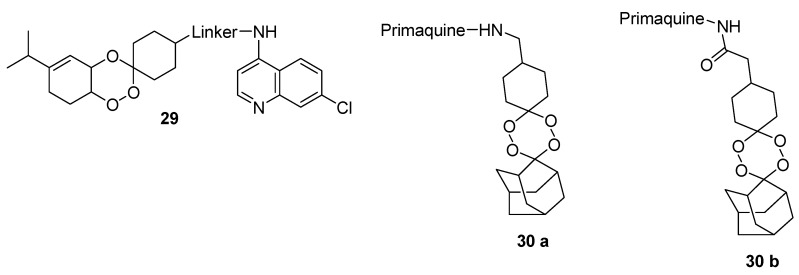
Quinoline-trioxolanes hybrid compounds.

Araújo et al. prepared a series of quinoline-trioxolanes hybrid compounds **29** (Figure 5). The antimalarial activity against chloroquine-sensitive and -resistant strain of *Plasmodium falciparum* revealed that the compounds were active in the low nanomolar range [47]. Trioxaquine DU1301 was prepared from 7-chloro-4-(2-aminoethylamino)quinoline and 1,2,4-trioxane [48]. In vivo studies showed that trioxaquines cured *Plasmodium* infected mice after administration orally at a dosage of 15 to 20 mg/kg of body weight/day for 4 days [48,49]. Trioxaquines inhibit heme aggregation within the parasite by forming unpolymerizable heme drug adducts and by stacking the quinoline fragment with heme [49]. Mirinda et al. prepared hybrid compounds containing1,2,4-trioxane or 1,2,4,5-tetraoxane and 8-aminoquinoline moieties **30** with antimalarial activity (Figure 5) [50]. The compounds were effective against exoerythrocytic and erythrocytic forms of malaria parasites. The compound with an amide linker between both moieties cleared blood-stage *P. berghei* infection in vivo [50]. 

### 4.4. Hybrid Compounds Containing Quinoline Derivatives and Antibacterial Agents

**Figure 6:**
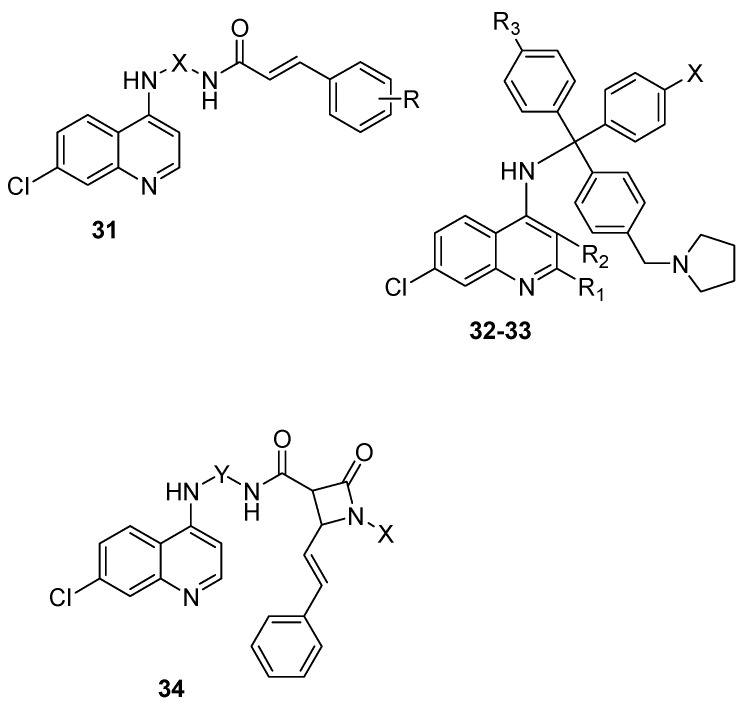
Hybrid compounds containing quinoline derivatives and antibacterial agents.

Quinoline-antibiotics have been developed using different antibacterial agents such as cinnamic acid. Hybrid compounds **31** containing a 4-amino-7-chloroquinoline and cinnamoyl scaffolds linked via an alkylamine chain have been reported to be a potent antimalarial in vitro (Figure 6) [51]. Hybrid compounds containing an aminobutyl chain were highly active against erythrocytic *P. falciparum* parasites [51]. Hybrid compounds **32** containing 4-aminoquinoline- and clotrimazole-based pharmacophores have been reported. The compounds interacted with free heme and *Plasmodium falciparum* heme metabolism. The compounds were effective in vitro against chloroquine-sensitive and -resistant *Plasmodium falciparum* strains. Some of the compounds were effective in vivo against *P. chabaudi* and *P. berghei* after oral administration [52]. Gemma et al. developed chloroquine-clotrimazole hybrid compounds **33** with antiplasmodial activity. The compounds displayed an IC_50_ value lower than 100 nM against all the selected strains used for the studies [53]. The hybrid compounds containing pyrrolidine and imidazole heterocycles exhibited a low degree of cross-resistance with chloroquine. However, hybrid compounds containing piperazine exhibited a high level of cross resistance, revealing the influence of the heterocyclic side chain on cross-resistance with chloroquine. In vivo studies on *P. berghei* mouse model further revealed that a single dose of the potent hybrid compound extended the mouse survival and increasing the dosage, enhanced the survival of all the mice [53]. Panda et al. developed quinine-quinolone hybrid compounds with antimalarial activity comparable in vitro with quinine with an IC_50_ between 12 and 207 nM [54]. Perez et al. prepared a class of cinnamic acid-4-aminoquinoline hybrid compounds. Replacing d-amino acids with natural L counterparts reduced the antiplasmodial activity of the compounds. Compounds with dipeptide linkers were effective against blood-stage *Plasmodium falciparum* in vitro and hemozoin formation [55]. The dipeptide linker influenced the antimalarial activity of the compounds [55]. In another research report by Perez et al., the blood and liver stage activities of the hybrid compounds was nullified when the 4-amino-7-chloroquinoline ring was replaced with a non-aromatic or an aromatic heterocycle. Replacement with primaquine cancelled the blood-stage activity of the compounds. These findings revealed the role of the 4-amino-7-chloroquinoline ring system in the dual-stage antimalarial activity [56]. Quinoline-lactam hybrid compounds have also been reported by some researchers. Singh et al. prepared 1,2,3-triazole tethered β-lactam-7-chloroquinoline bifunctional hybrids [57]. The *N*-substituent of the β-lactam ring and bis-triazole at the C-3 position enhanced the antimalarial activity of the compounds and inhibited the growth of *P. falciparum*. The IC_50_ values of the potent compounds varied between 80 and 94 nM comparable to chloroquine [57]. Raj et al. prepared a class of quinoline-β-lactam hybrid compounds **34** in which the antimalarial activity was influenced by the linker, N-1 substituent of the β-lactam ring and the alkyl chain length. Most of the compounds were not effective against *Plasmodium falciparum* W2 strain when compared to chloroquine [58,59]. The urea containing compounds displayed IC_50_ values between 42 and 193 nM whereas compounds with oxalamido group IC_50_ values ranged between 34 and 120 nM. The *N*-aryl substituent with shorter chain length was more effective than cyclohexyl substituent with longer alkyl chain lengths (Figure 6) [59]. 

### 4.5. Quinoline-Mercaptopurine Hybrid

Hybrid compounds containing quinolines and anticancer drugs with good antimalarial activity have been reported. De Souza et al. prepared quinoline-mercaptopurine hybrid compounds [60]. The compounds were not cytotoxic at a concentration of 100 μg/mL. In vivo evaluation revealed that the compound was effective against *P. berghei* with over 70% inhibition of parasite multiplication at a dosage of 5 mg/kg/day over a period of 4 days [60]. 

### 4.6. Quinoline-Pyrimidine Hybrid Compounds

**Figure 7:**
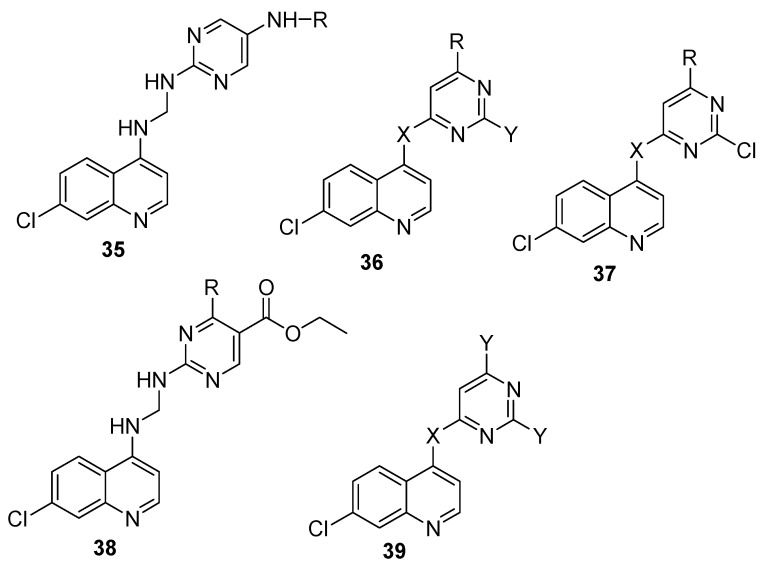
Quinoline-pyrimidine hybrid compounds.

Quinoline-pyrimidine hybrid compounds have been reported by several researchers (Figure 7). Tripathi et al. prepared 4-aminoquinoline-pyrimidine hybrids with modified anilines [61]. The hybrids displayed excellent 11-fold higher in vitro antiplasmodial activities against chloroquine-sensitive D6 and chloroquine-resistant W2 strains of *P. falciparum* with an IC_50_ = 0.033 μM when compared to chloroquine with an IC_50_ = 0.370 μM against the resistant strain. The good binding interactions of the compounds with monomeric and dimeric heme suggested that their mode of action is by heme-detoxification. The potent compounds displayed good in silico docking interactions within the active site of plasmodial dihydrofolate reductase (DHFR) enzyme [61]. Manohar et al. prepared a series of 4-aminoquinoline-pyrimidine hybrids [62]. In vitro antimalarial activity showed that some of the compounds exhibited good antimalarial activity when compared to chloroquine or pyrimethamine. The compounds containing 4-aminoquinoline and *N*-methylpiperazine moieties with two to five carbon spacers were 6–8 times more effective compared to chloroquine and between 1.6 and 2.0 times more effective compared to pyrimethamine against the D6 strain. The compounds containing 4-aminoquinoline and *N*-methyl piperazine or *N*-ethyl piperazine moieties were 6–26 times more effective against chloroquine resistant strain when compared to chloroquine [62]. The point of attachment of the spacer to the pyrimidine nucleus did not influence its antimalarial activity. Kumar et al. prepared a series of novel 4-aminoquinoline-pyrimidine hybrids **35** effective in vitro against both chloroquine-sensitive and chloroquine-resistant strains [63]. Some of the compounds suppressed parasitemia significantly in vivo [63]. Increase or decrease in the length of the carbon chain linker had no effect on the antimalarial activity of the compounds [64]. However, compound with phenyl ring at the pyrimidine nucleus exhibited antimalarial activity in the range of 0.049–0.074 μM against D6 strain and 0.075–0.142 μM against W2 strain. The compound with ethylene linker was very effective against chloroquine-sensitive strain while compounds with butylene linker was active against chloroquine resistant strain. In vivo antimalarial activity via oral administration in *P. berghei*-mouse malaria model once daily showed 17.85%, 37.62% and 96.42% parasite suppression, respectively, at 11.1, 33.3 and 100 mg kg^−1^ doses on day 5 as compared to 100% suppression displayed by chloroquine [64]. Shah et al. prepared a class of quinoline-pyrimidine linked calixarene derivatives with selected quinolines such as 5-, 8-amino quinoline, 4-amino-3-methyl quinoline, 8-hydroxyquinoline and 2-aminopyrimidine [65]. Compounds containing 2-aminopyrimidine and 8-hydroxyquinoline exhibited good antimalarial activity with IC_50_ values of 0.073 and 0.043 µg/mL, respectively, comparable with chloroquine [65]. The electronegativity of the quinoline scaffold enhanced the antimalarial activity of the compounds [65]. Kaur et al. reported a series of hybrids comprising of 5-cyanopyrimidine and quinoline moiety **36** [66]. Hybrid bearing *m*-nitrophenyl substituent at C-4 of pyrimidine displayed a four-fold antiplasmodial activity with IC_50_ = 56 nM against the CQ^R^ (Dd2) strain than chloroquine [66]. Compounds with a four-methylene spacer exhibited good antiplasmodial activity when compared to compounds with a three-methylene spacer. Compounds with piperazinyl linker resulted in a complete loss of antiplasmodial activity, which is attributed to the steric restrictions hindering a face-to-face stacking of the quinoline unit with heme and ineffective inhibition of the heme polymerization to hemozoin [66]. Thakur et al. reported a class of 4-aminoquinoline-pyrimidine hybrids conjugated via piperazine **37** with in vitro antimalarial activity against chloroquine sensitive and resistant strains of *Plasmodium falciparum* [67]. Most of the compounds displayed IC_50_ values below 1 µM for both strains of *Plasmodium falciparum*. The potent compounds exhibited IC_50_ values in the range of 0.13–0.14 μM. A decrease in the size of the ring from piperidine to pyrrolidine resulted in a three to four-fold improvement in the antimalarial activity. The presence of primary amines in the pyrimidine ring of the hybrids improved the antimalarial activity with IC_50_ values ranging between 0.13 and 0.39 µM against D6 strain. Replacing primary and secondary amines with substituted anilines decreased the antimalarial activity of the compounds in selected compounds [67]. Singh et al. prepared 2-aminopyrimidine-based 4-aminoquinolines **38** [68]. They exhibited in vitro antiplasmodial activity against drug-sensitive and drug-resistant chloroquine strains of *Plasmodium falciparum*. The antimalarial activity of 5-isopropyloxycarbonyl-6-methyl-4-(2-nitrophenyl)-2-[(7-chloroquinolin-4-ylamino)butylamino] pyrimidine was 56 fold less than that of chloroquine, with an IC_50_ of 3.6 nM against a chloroquine-resistant strain [68]. Pretorius et al. prepared quinoline–pyrimidine hybrids **39** by nucleophilic substitution of quinoline and pyrimidine moieties [69]. In vitro evaluation against the D10 and Dd2 strains of *Plasmodium falciparum* revealed that the compounds were effective against both strains. Compound with piperazine linker was the most active with IC_50_ of 0.157 nM comparable to an equimolar fixed combination of chloroquine and pyrimethamine against both strains [69].

### 4.7. Quinoline–Sulfonamide Hybrids

**Figure 8:**
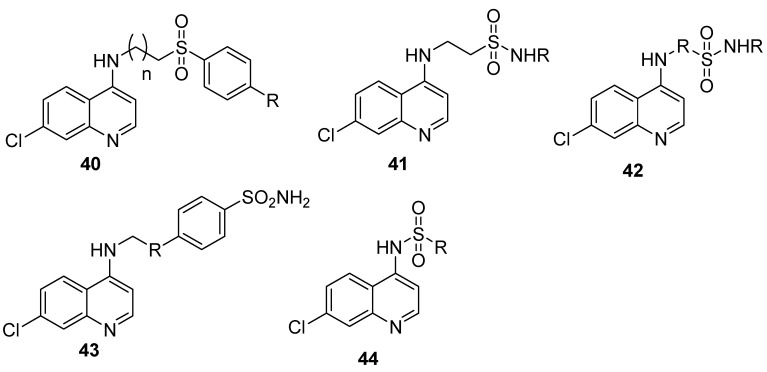
Quinoline-sulfonamide hybrids.

Hybrid compounds containing quinoline and sulphonamide moieties have been reported as potential antimalarials (Figure 8). Pinheiro et al. prepared quinoline-sulfonamide hybrids **40** containing a linker group connecting arylsulfonamide moieties to the aminoquinoline molecule. The compounds displayed good schizonticidal blood activity in vitro with IC_50_ values ranging from 0.05 to 1.63 μM. Some of the compounds IC_50_ values ranged from 0.05 to 0.40 μM which were lower than that of chloroquine and sulfadoxine. These later compounds inhibited *Plasmodium berghei* parasitemia by 47% and 49% on day 5 after mice inoculation [70]. Verma et al. prepared quinoline-sulfonamide hybrids **41** with antimalarial activity of IC_50_ 0.05 and 0.01 μM against 3D7 and 0.41 and 0.36 μM against K1, respectively. The compounds inhibited the formation of hemozoin significantly [71]. Soares et al. prepared quinoline-sulfonamide hybrids **42** with hydrazine or hydrazide linkers [72]. The compounds were effective against chloroquine-sensitive, 3D7 and chloroquine-resistant strains W2. The hydrazine derivative compound was effective in vivo activity against *P. berghei*. The rate of reduction of blood parasites at a dose of 10 mg/mL was higher compared to chloroquine [72]. Fisher et al. prepared 7-chloro-4-aminoquinoline-sulfonamide hybrid compounds **43**. The antimalarial activity of the hybrid compounds against chloroquine-sensitive, 3D7 and chloroquine-resistant, Dd2 *Plasmodium falciparum* strains was however low when compared to chloroquine [73]. Salahuddin et al. prepared 4-aminochloroquinoline-sulfonamides **44** with good antimalarial activities [74]. Some of the compounds displayed antimalarial activity against the chloroquine-resistant, FCR-3 strain of *Plasmodium falciparum* with IC_50_ values less than 2 μM. The absence of haemolysis of the uninfected human erythrocytes suggested that the compound entered the parasite, inhibiting parasite growth without interfering with the integrity of the red blood cell membrane. The potent compound, (4-{[4-(7-chloroquinolin-4-yl)piperazin-1-yl]sulfonyl}-*N*-acetylaniline) was sevenfold less active compared to quinine [74]. 

### 4.8. Hybrid Compounds Containing Quinoline and Other Ring Systems

**Figure 9:**
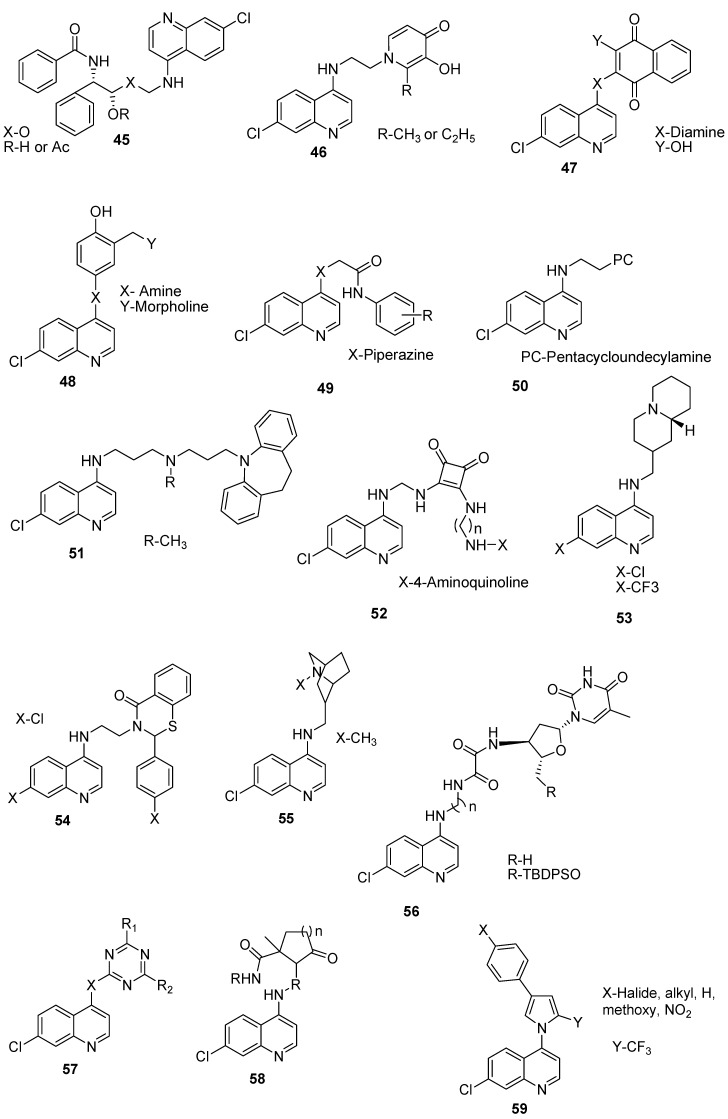
Hybrid compounds containing quinoline and other ring systems.

Hybrid compounds containing quinoline and other ring systems with good antimalarial activity have been prepared by several researchers (Figure 9). Njogu et al. prepared hybrid compounds **45** containing quinoline and (2*R*,3*S*)-*N*-benzoyl-3-phenylisoserine moieties linked by an ester, amide, or triazole functionalities [75]. The compounds were effective against W2 strain of *P. falciparum*. However, their antiplasmodial activities were lower than chloroquine. Although hybridization of quinoline with (2*R*,3*S*)-*N*-benzoyl-3-phenylisoserine moiety did not enhance the in vitro antiplasmodial activities of the compounds, the pharmacological activity of the 4-amino-7-chloroquinoline moiety was retained [75]. Andayi et al. prepared 4-aminoquinoline-3-hydroxypyridin-4-one hybrid analogues **46** active against chloroquine-resistant *P. falciparum*. Some of the hybrids were potent inhibitors of β-hematin formation when compared to chloroquine which increased with increase in the lipophilicity of the hybrid compounds. Antiplasmodial activity of the hybrid molecules was dependent on β-hematin inhibition [76]. Kashyap et al. prepared quinolone-based hybrids **47** containing 7-chloro-4-aminoquinoline and 3-amino-1,4-naphthoquinone scaffolds linked via diamines [77]. In vitro studies against chloroquine-sensitive RKL-2 and chloroquine-resistant RKL-9 strains of *Plasmodium falciparum* showed that the hybrids were effective against RKL-2 and RKL-9 strains with IC_50_ values ranging between 0.391 and 1.033 µg/mL and 0.684 to 1.778 µg/mL, respectively, at the same dose. The hybrid compound having a diaminoethyl moiety exhibited good activity against both the sensitive and resistant strains of *P. falciparum* when compared to compounds with diaminopropyl and 2,3-diaminophenyl moieties which were less active against both strains of *P. falciparum* [77]. The drug-likeness studies further suggest that the drug-likeness behavior of the hybrids enhanced their membrane permeability and interaction with the receptor [77]. Singh et al. prepared 4-anilinoquinoline-Mannich base drugs **48** [78]. The compounds were effective in vitro against chloroquine-sensitive 3D7 and RKL-2 strain of *P. falciparum*. However, the compound containing diphenylamine was not effective against the strains of *P. falciparum* when compared to compound with a morpholine moiety that displayed promising results in vivo in Swiss mice model infected with rodent malaria parasite *Plasmodium yoelii* (strain N-67) [78]. Inam et al. prepared 2-[4-(7-chloroquinolin-4-yl)piperazin-1-yl]acetamide compounds **49** with antimalarial activity. The compounds’ in vitro growth inhibition of *P. falciparum* IC_50_ ranged between 0.30 and 33.52 μM. One of the compounds was effective in inhibiting the formation of β-haematin [79]. Lödige and Hiersch prepared primaquine-chloroquine hybrid compounds with different linkers such as an elongated piperazine diamide, diamine linker bond and an aromatic-based linker [25]. The hybrid compound of primaquine-chloroquine in a 1:1 ratio reduced the concentration of the liver stage parasites of *P*. *berghei* significantly when compared to free primaquine. Hybrid compound containing primaquine and chloroquine via piperazine-linker with amide functionality reduced the number of liver stage parasites of *P*. *berghei* when compared to the hybrid with amine functionality. Hybrid compounds with two chloroquine moieties were not effective against the parasites. In vitro evaluation against strains of against blood stages of *P*. *falciparum* namely: 3D7, Dd2 and K1 revealed that the hybrid compound of primaquine–chloroquine in a 1:1 ratio showed lower activity than chloroquine but was effective at a lower concentration when compared to primaquine and chloroquine. The hybrid compound showed the best activity which was five times higher against the K1 strain compared to 3D7 and Dd2 strain, respectively. This is attributed to the resistance-reversing effect of primaquine. All other hybrid compounds prepared showed good activities against all strains however, they were slightly less active compared to the primaquine-chloroquine in a 1:1 ratio. Their low activity is attributed to factors such as lack of a strong basic center such as the tertiary nitrogen atom, linkage properties, the pharmacophore ratio and the distance between the pharmacophore moieties. The hybrid molecules hindered the development of gametocytes and the linkage moiety, the basicity, and the pharmacophore ratio did not influence their activity against gametocytes [25].

Joubert et al. prepared hybrid molecules **50** containing pentacycloundecylamine moiety, a reversal agent conjugated to an aminoquinoline molecule [80]. They were synthesized by conjugating the Cookson’s diketone with selected tethered 4-aminoquinoline compounds, resulting in a class of carbinolamines and subsequent imines which was followed by transannular cyclization. The hybrid antiplasmodial IC_50_ values were in the ranges of 3.74–17.6 nM and 27.6–253.5 nM, respectively, against chloroquine-sensitive (D10) and chloroquine-resistant strains (Dd2) strain of *Plasmodium falciparum*. The activity of the hybrid molecules was reduced significantly in the chloroquine-resistant strain. The compound with a chain length of two carbon atoms exhibited optimum antiplasmodial activity. Increasing the carbon-chain length between the aminoquinoline and pentacyclo-undecylamine moeities reduced the antiplasmodial activity significantly in the resistant strain [80]. Burgess et al. prepared a hybrid molecule, *N*′-(7-chloroquinolin-4-yl)-*N*-,[3-(10,11-dihydrodibenzo[*b*,*f*]azepin-5-yl)propyl]-*N*-methylpropane-1,3-diamine (**51**) from 4-aminoquinoline and imipramine [81]. The IC_50_ value of the compound was 2.9 nm against D6 strain and 5.6 against Dd2 strain. The IC_50_ values were low when compared to chloroquine, suggesting that attaching the imipramine moiety enhanced the activity of the aminoquinoline. In vivo evaluation by oral administration in mice showed that 64 mg/kg/day inhibited 99% growth of *P. chabaudi* after 4 days. The effect was dose-dependent and lowering the dosage resulted in no inhibition of the *Plasmodium* growth. Higher dose was required in vivo due to the lipophilic behaviour and the metabolism of the compound in the liver [81]. In another research report by Burgess et al. selected linkers were used between the chloroquinoline ring and imipramine moieties [82]. The compounds exhibited significant activity against both D6 and Dd2 *P. falciparum* malaria strains. 

Hybrid compounds with a dibenzylamino moiety were effective against chloroquine-resistant and -sensitive strains of *P. falciparum* when compared to those with diphenylamino group. The effect of the length of the linker on the antimalarial activity of the hybrids was not significant [82]. Ribeiro et al. prepared hybrid compounds 52 containing a squaric moiety conjugated with aminoquinolines [83]. Some of the compounds exhibited greater in vitro potency compared to chloroquine against chloroquine-resistant *Plasmodium falciparum*. They were also non-toxic. Sparatore et al. prepared hybrid compounds 53 containing 4-aminoquinoline with either a piperidine or a pyrrolidine ring. The compounds were very basic and were active against chloroquine resistant strains, with IC_50_ values ranging between 21 and 23 nM [84]. The activity of the compounds decreased with increase in the length of the linker between both moieties. The compounds were active against a chloroquine-sensitive strain with IC_50_ values ranging from 24.2 to 102.4 nM [84]. Solomon et al. prepared hybrid compounds 54 containing 4-aminoquinolines and thiazolidin-4-one, thiazinan-4-one or 2,3-dihydrobenzo[*e*][1,3]-thiazin-4-one. The compounds formed a complex with hematin, thereby inhibiting the formation of β-hematin [85]. Khan et al. prepared hybrids 55 containing isoquinuclidine and quinoline moieties [86]. The compounds with a methylene amine bridge exhibited significant antimalarial activity against both chloroquine-susceptible D6 (2-fold) and the chloroquine-resistant W2 strains of *P. falciparum* (30-fold). The compound in which the terminal amine side chain is replaced with a less bulky methyl group was as potent as chloroquine against the D2 clone and about 7-fold more potent against the W2 clone [86]. Cornut et al. prepared compounds 56 containing fluoroalkylated γ-lactams and 4-aminoquinoline. The most potent compounds was effective against chloroquine-sensitive strain 3D7 with IC_50_ values of 26 and 19 nM and against multi-drug-resistant strain W2 with IC_50_ values of 49 and 42 nM, respectively [87]. Sunduru prepared compounds 57 containing 4-aminoquinolines and oxalamide and triazine derivatives with antimalarial activities [88]. Compounds containing a triazine were most active against chloroquine-sensitive strain 3D7 of *Plasmodium falciparum* with an IC_50_ of 5.23 ng/mL and 7.88 ng/mL. The compound with an oxalamide moiety inhibited 70.45% on day 4 against chloroquine-resistant strain N-67 of *Plasmodium yoelii* in vivo [88]. Musonda et al. developed a class of hybrids 58 containing 4-aminoquinoline γ- and δ-lactams [89]. The most potent compound was effective against a chloroquine-resistant W2 strain of *Plasmodium falciparum* with an IC_50_ of 0.096 μM [89]. Compounds with six-membered lactam ring were more effective than the five-membered ring [89]. Cunico et al. evaluated antimalarial activity of chloroquine-pyrazole hybrids 59 prepared from the reaction of 1,1,1-trifluoro-4-methoxy-3-alken-2-ones with 4-hydrazino-7-chloroquinoline [90]. The IC_50_ values ranged between 1.39 and 1.69 mg/mL for the most potent compounds. They were effective against *P. berghei* in mice in vivo. However, the (pyrazol-1-yl)chloroquine compounds were inactive, indicating that the hydroxyl functionality in the pyrazole ring was important for antimalarial activity [90].

## 5. Lipinski Rules

Although hybrid compounds are potential molecules which can overcome drug resistance, some of them have a large size which can limit their oral bioavailability. Lipinski’s rule, also known as the “rule of five”, was proposed for drug-likeness, which states that poor permeability of a compound will occur if the hydrogen-bond donors are greater than five, if the molecular mass is greater than 500 with calculated log *P* greater than 5 and if the sum of nitrogen and oxygen atoms is greater than 10 [91]. However, it is important to mention that this rule is not applicable to natural products and drugs which are substrates of biological transporters such as proteins and antibodies [91,92]. The rule indicates the significance of absorption, distribution, metabolism and elimination (ADME) and physico-chemical properties useful for the advancement of drug discovery process [91]. It is an important rule used in the pharmaceutical industry to determine the oral bioavailability of small drug molecules [91]. There are few research reports on the drug-likeness score for quinoline-based hybrid compounds with antimalarial activity. In hybrid compounds containing 7-chloro-4-aminoquinoline and 3-amino-1,4-naphthoquinone scaffolds reported by Kashyap et al. most of the compounds exhibited good drug-likeness scores in the range of 0.07–0.78 based on Lipinski’s rule of five. The result confirmed that the hybrid compounds have good bioavailability and can penetrate the parasite cell to attain significant intracellular accumulation, resulting in a good antimalarial activity [77]. Compounds with bulky aromatic group linkers did not obey Lipinski’s rule of five. Their antimalarial activity was reduced when compared to compounds with small alkyl linkers [77]. Sharma et al. reported similar findings in which synthesized hybrid compounds with good drug-likeness properties exhibited good antimalarial activity [93]. Compounds with smaller alkyl groups exhibited superior antimalarial activity than compounds with bulky moieties [93]. Maurya et al. prepared 4-aminoquinoline-pyrimidine hybrid compounds that obeyed Lipinski’s rule of five. Those that obeyed the rule also exhibited good antimalarial activity [2]. The compounds exhibited potent antimalarial activity against strains of *P. falciparum* (D6 and W2) with IC_50_ values 0.038–0.044 μM which was equipotent to artemisinin, a potent antimalarial drug with IC_50_ = 0.045 μM [2]. Salahuddin et al. prepared 4-aminochloroquinoline-sulfonamides hybrid compounds and the most potent compounds against *P. falciparum* had log *P* values nearly equal to quinine and chloroquine [74]. Rojas et al. prepared hybrid compounds containing 4-aminoquinoline and 2-imino-thiazolidin-4-one or 1*H*-pyrrol-2,5-dione moiety. The drug-likeness score was in a range of 0.16 to 0.87 based on Lipinski’s rule of five and comparable to chloroquine with a score of 0.67 [94]. Kumar et al. reported hybrid compounds which obeyed Lipinski’s rule of five which further suggested good oral bioavailability influenced by the flexibility of the compounds [95]. On the other hand, quinoline-based hybrid compounds have large sizes. However, some of them have been reported to obey Lipinski’s rule of five which further confirm that they are potential antimalarials. 

## 6. Conclusions

Hybrid compounds with antimalarial activity containing quinoline are cost effective, with reduced risk of drug-drug interactions. In the design of the hybrid compounds, the linker influences the pharmacokinetic behaviour of the drugs. There are several reports of hybrid compounds containing quinoline with good antimalarial activity. Artemisinin-quinoline hybrid compounds exhibited good antimalarial activity when compared to the individual drugs. However, some of the hybrid compounds were found to be less effective compared to the individual drugs. The chain length between the artemisinin and quinoline moieties influenced the antimalarial activity of the compounds. Quinoline-ferrocene analogues act as resistance-reversing agents, thereby inhibiting resistance of the *Plasmodium* parasites. Their ability to overcome resistance is attributed to their lipophilicity. However, hybrids with rigid linkers were not active biologically when compared to hybrids with flexible linkers. Quinoline-trioxolanes inhibit heme aggregation within the parasite, either by forming unpolymerizable heme drug adducts or by stacking the quinoline fragment with heme. The nature of the linker has an influence on the antimalarial activity of the compounds in vitro and in vivo. Hybrid compounds containing a quinoline moiety and antibiotics or anticancer drug with good antimalarial activity were also reported. They were effective against blood and liver stage malaria with high percentages of inhibition of parasite multiplication. The design of the compounds influenced the antimalarial activity. Selected 4-aminoquinoline-pyrimidine hybrids are potent antimalarials and some of them were 6–26 times more effective compared to chloroquine. The mode of action of the compounds is by heme-detoxification. The length of the chain linker did not have effect on the antimalarial activity of some of the compounds, however, they were selective towards either chloroquine-sensitive strain or chloroquine resistant strain depending on the nature of the linker. Hybrid compounds with piperazinyl linker nullified the antiplasmodial activity which is attributed to the steric restrictions hindering a face-to-face stacking of the quinoline unit with heme and ineffective inhibition of the heme polymerization to hemozoin. Quinoline-sulfonamide hybrids exhibit good antimalarial activity but most of them were less active when compared to chloroquine. They acted by inhibiting parasite growth without interfering with the integrity of the red blood cell membrane. To date, the structural modifications in heterocyclic systems have provided advancements in the development of drugs for the treatment of malaria, however, some heterocyclic systems have not been explored, which could provide additional new pharmacophores and drugs for the treatment of malaria. There is no doubt that the continuous advancement in the development of quinoline heterocyclic compounds will give rise to potent antimalarials to overcome drug resistance which is common with the currently used antimalarials.
